# Supplementary material for: Evaluation of variant calling for cpn60 barcode sequence-based microbiome profiling
Source: PLoS One. 2020 Jul 9;15(7):e0235682. doi: 10.1371/journal.pone.0235682 (PMC7347135; doi:10.1371/journal.pone.0235682)
Supplement: S1 Fig — Various lengths starting from the 5’ end of the cpn60 UT were extracted from a bacteria-only version of cpnDB_nr. Alignments were conducted with clustalw2 and a similarity table was formed with dnadist from the phylip package. Mean and median percent identities were determined for each set of alignments. (PDF) [file pone.0235682.s001.pdf]

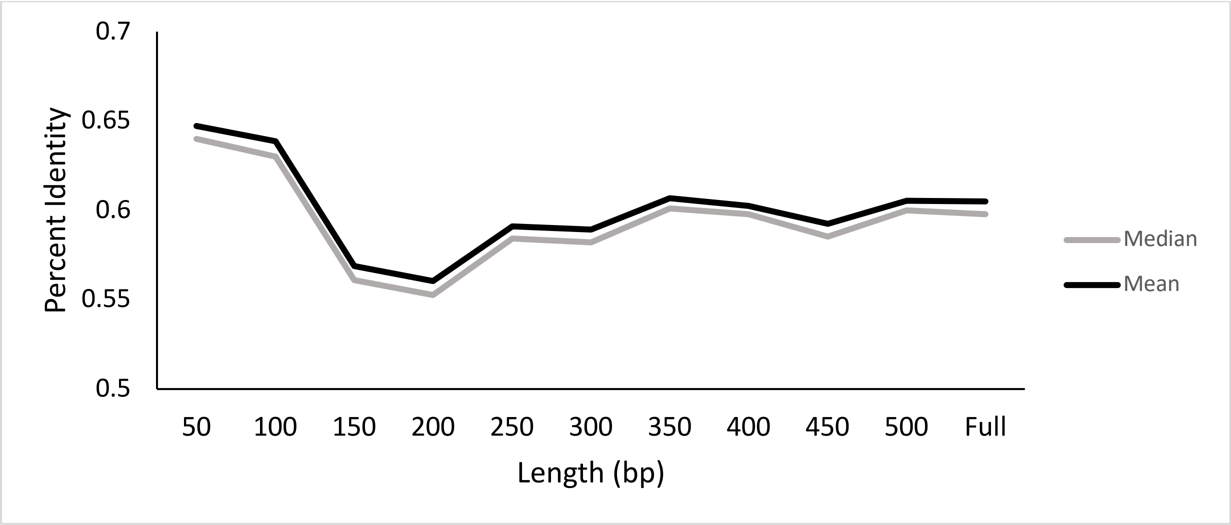

Figure S1: Multiple All vs. All bacterial cpnDB_nr alignments. Various lengths starting from the 5' end of the *cpn*60 UT were extracted from a bacteria-only version of cpnDB_nr. Alignments were conducted with clustalw2 and a similarity table was formed with dnadist from the phylip package. Mean and median percent identities were determined for each set of alignments.
