# Supplementary material for: Evaluation of variant calling for cpn60 barcode sequence-based microbiome profiling
Source: PLoS One. 2020 Jul 9;15(7):e0235682. doi: 10.1371/journal.pone.0235682 (PMC7347135; doi:10.1371/journal.pone.0235682)
Supplement: S1 Table — (PDF) [file pone.0235682.s002.pdf]

Table S1: Illumina adapted *cpn*60 PCR primer sequences.

Primer	Sequence (5'-3')
M279	TCG TCG GCA GCG TCA GAT GTG TAT AAG AGA CAG GAI III GCI GGI GAY GGI ACI ACI AC
M280	GCT TCG TGG GCT CGG AGA TGT GTA TAA GAG ACA GYK IYK ITC ICC RAA ICC IGG IGC YTT
M1612	TCG TCG GCA GCG TCA GAT GTG TAT AAG AGA CAG GAI III GCI GGY GAC GGY ACS ACS AC
M1613	GCT TCG TGG GCT CGG AGA TGT GTA TAA GAG ACA GCG RCG RTC RCC GAA GCC SGG IGC CTT
